# Relationship between hamstring strength and hop performance at 8 and 12 months after ACL reconstruction with hamstring tendon autografts

**DOI:** 10.1186/s13102-024-00923-4

**Published:** 2024-06-18

**Authors:** Johan Högberg, Jakob Lindskog, Axel Sundberg, Ramana Piussi, Rebecca Simonsson, Kristian Samuelsson, Roland Thomeé, Eric Hamrin Senorski

**Affiliations:** 1 Sportrehab Sports Medicine Clinic, Stampgatan 14, Gothenburg, SE-411 01 Sweden; 2 Sahlgrenska Sports Medicine Center, Gothenburg, Sweden; 3 Unit of Physiotherapy, Department of Health and Rehabilitation, Institute of Neuroscience and Physiology, Sahlgrenska Academy, University of Gothenburg, Box 455, Gothenburg, SE-405 30 Sweden; 4 Capio Ortho Center, Drakegatan 7A, Gothenburg, SE-412 50 Sweden; 5 Department of Orthopaedics, Institute of Clinical Sciences, Sahlgrenska Academy, University of Gothenburg, Gothenburg, Sweden; 6 Department of Orthopaedics, Sahlgrenska University Hospital, Mölndal, Sweden

**Keywords:** Anterior cruciate ligament, Hamstring tendon autograft, Isokinetic dynamometer, NordBord, Hop performance

## Abstract

**Background:**

The relationship between hamstring strength and hop performance after anterior cruciate ligament (ACL) reconstruction with hamstring tendon (HT) autografts has not been well elucidated. The aim was to investigate the relationship between eccentric hamstring strength, assessed with the NordBord, and concentric hamstring strength, assessed with the Biodex, with hop performance at 8 and 12 months after ACL reconstruction.

**Methods:**

Registry study. Patients ≥ 16 years who had undergone primary ACL reconstruction with HT autograft, followed by muscle strength and hop tests at 8 and 12 months were included. Correlations of the relative hamstring strength (Nm/kg or N/kg) and limb symmetry index (LSI) with hop performance were analyzed. Pearson’s correlation coefficient, and coefficient of determination (r^2^) were used for statistical analysis.

**Results:**

A total of 90 patients were included, of which 48 (53%) were women. The mean age at ACL reconstruction was 27.0 ± 8.0 years. Relative hamstring strength had significant positive correlations with hop performance, ranging from *r* = 0.25–0.66, whereas hamstring strength LSI had significant positive correlations which ranged from *r* = 0.22–0.37 at 8 and 12 months after ACL reconstruction. At 12 months, the relative hamstring strength in the Biodex explained 32.5–43.6% of the hop performance in vertical hop height, hop for distance relative to height, and the total number of side hops, whereas the relative hamstring strength in the NordBord explained 15.2–23.0% of the hop performance.

**Conclusion:**

The relative hamstring strength in the Biodex test explained 32.5–43.6% of the hop performance, whereas the relative hamstring strength in the NordBord explained 15.2–23.0%. Thus, our findings suggest that relative hamstring strength, especially in the hip-flexed position may be a better indicator of hop performance at 8 and 12 months after ACL reconstruction in patients treated with HT autograft.

**Supplementary Information:**

The online version contains supplementary material available at 10.1186/s13102-024-00923-4.

## Background

The hamstring tendon (HT) autograft is globally the most frequently used autograft for surgical reconstruction of a ruptured anterior cruciate ligament (ACL) [1]. More specifically, the semitendinosus tendon is used either alone or in combination with the gracilis tendon to provide a sufficient graft diameter to ensure adequate strength and stiffness of the autograft [2]. The harvest of a HT for the use as an autograft is associated with consequences such as retraction and atrophy of the semitendinosus muscle with a longer and thinner tendon which also inserts more proximally compared to the uninjured side [3, 4]. However, whether subsequent differences in the morphological properties of the hamstrings after tendon harvesting affect knee function after ACL reconstruction with HT autografts is yet to be determined.

The hamstring muscle group is considered important for knee stability, suggested to reduce excessive strain on the ACL induced by the quadriceps during strenuous knee activities [5], where the medial part of the hamstrings (including the semitendinosus) provides medial stability during stepping maneuvers [6]. The assessment of the hamstring strength recovery after ACL reconstruction is commonly considered in relation to the non-injured side, i.e., limb symmetry index (LSI) [7], where achieving ≥ 90% LSI has previously been considered as “recovered” [8]. However, the investigation of hamstring strength recovery depends on how hamstring strength is assessed. Comparisons between the eccentric NordBord test (Vald Performance NordBord, Version 1.0, Australia), based on the exercise Nordic hamstring, with the ‘gold standard’ seated isokinetic concentric knee flexion strength test in a Biodex dynamometer (Biodex Medical Systems, Inc., Shirley, NY, USA) have displayed significantly lower LSI values in the NordBord test during the first year, at mid-term (2 and 5 years), and at long-term follow-up after ACL reconstruction with HT autograft (14.4 years) [9–11]. At 1 year after ACL reconstruction, when patients typically are advised to return to sport [12, 13], the passing rate of ≥ 90% in LSI in the NordBord test is reportedly low with 41%, compared to 73% in the Biodex test [9]. The relevance of a persistent hamstring strength deficit in the NordBord test is, however, unknown. No significant correlations were observed between hamstring LSI and patient-reported outcomes (PROs) with regard to perceived knee function during the first year after ACL reconstruction with HT autografts [14]. In contrast, significant positive correlations between peak force in relation to body weight in NordBord and perceived knee function have been reported at 4 and 8 months, accounting for 9–14% of the variance in perceived knee function [14]. Furthermore, Ogborn et al. [10] reported a significant association between peak force in the NordBord test and the results of the ACL-QOL questionnaire, accounting for 23% of the variance in perceived knee function. The peak force in relation to the body weight in the NordBord has also displayed significant positive moderate correlations with the total number of side hops in a side hop test for 30 seconds (s) at 2 and 5 years after ACL reconstruction [11]. Collectively, the relevance of hamstring strength deficits in the NordBord test for patients treated with ACL reconstruction with HT autografts is mainly based on associations with PROs, and the relationship with knee function during the first year is lacking. Hop tests are commonly employed to assess patients’ knee function as a part of the informed decision-making prior to returning to sport [15]. Further investigation of hamstring strength deficits in the NordBord test for patients treated ACL reconstruction with HT autografts and knee function such as hop performance may provide better insight into the relevance of hamstring strength assessed in the NordBord test.

The purpose of this study was to investigate the relationship between hamstring strength assessment in the eccentric NordBord and concentric Biodex tests with hop performance at 8 and 12 months in patients after ACL reconstruction treated with HT autograft.

## Methods

### Study design

The REporting of studies Conducted using Observational Routinely Collected health Data (RECORD) statement was used to guide the writing of the present study [16]. This study was a registry study based on data from a local rehabilitation registry (Project ACL) in Gothenburg, Sweden. Ethical approval was obtained from the Swedish Ethical Review Authority (2020–02501).

### Setting

Project ACL is open for individuals who have sustained an ACL injury, regardless of the time that has passed since the injury and treatment choice, that is, rehabilitation with or without reconstruction. Upon participation in Project ACL, patients are asked to respond to PROs and perform muscle strength tests for the quadriceps and hamstring muscle groups and hop performance according to a standardized schedule starting from 10 weeks after ACL injury/reconstruction, followed by 4, 8, 12, 18, 24, and 60 months, and then every fifth year. Participation is voluntary and can be withdrawn at any time. Informed consent is obtained from the patients at the time of inclusion in the Project ACL.

### Patient selection

For the present study we used the following inclusion criteria: 1) primary ACL reconstruction with HT autograft, 2) ≥ 16 years old at the time of ACL reconstruction, 3) had performed isokinetic muscle strength testing, the NordBord test, and hop tests at 8 and 12 months after ACL reconstruction, and 4) rated ≥ 6 on the Tegner activity (Tegner) scale preinjury. Patients who had two or more ACL injuries, and who did not have complete data (all muscle strength and hop tests) for muscle strength and hop performance at 8 and 12 months after ACL reconstruction were excluded. The modified Tegner scale is used in Project ACL, which aims to describe the level of strenuous knee activity in which an individual commonly participates, ranging from 1 to 10, with higher values indicating more strenuous knee activities [17]. A rating ≥ 6 on the Tegner scale reflects participation in pivoting sports, that is, participating in knee-strenuous activities. The choice of ≥ 6 on the Tegner Activity Scale was to ensure an active study population. A high test-retest reliability with intraclass coefficient (ICC) value of 0.8 and an acceptable floor and ceiling effects have been reported for the Tegner scale [18].

### Muscle strength tests

**Fig. 1:**
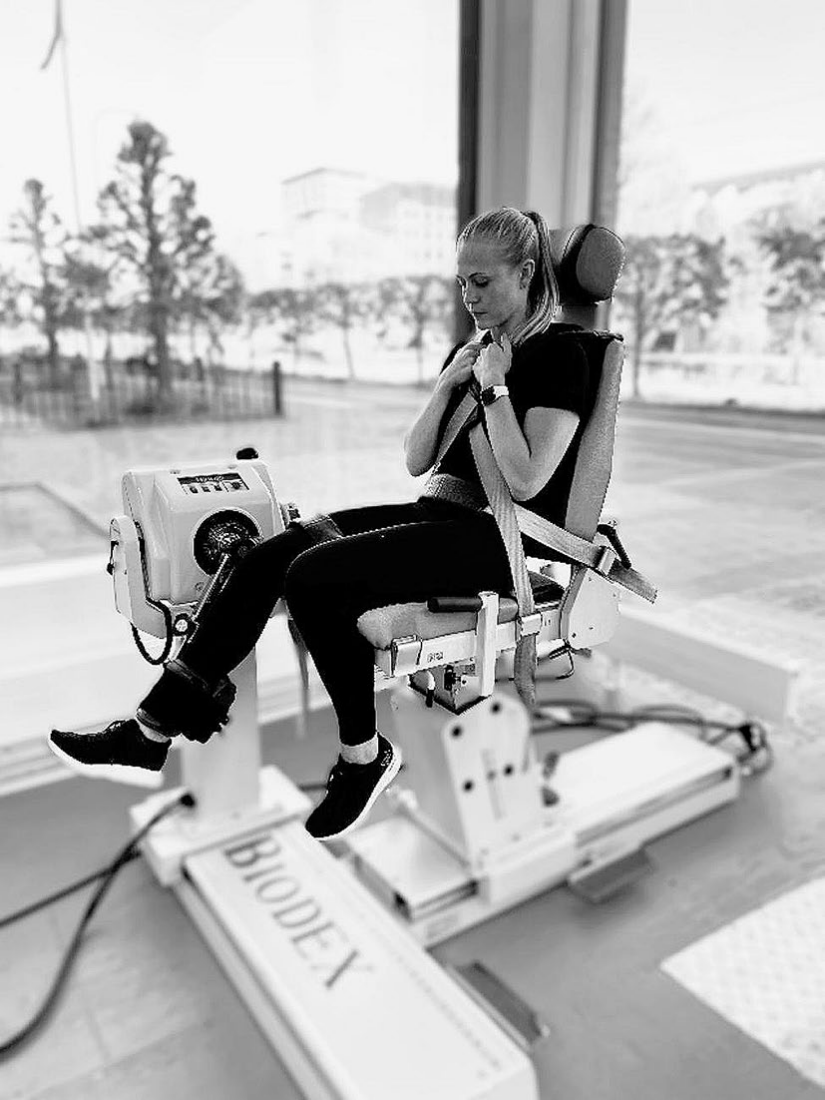
The isokinetic concentric seated leg-curl test performed in the Biodex

Two different methods were used to assess the maximal hamstring muscle strength. First, isokinetic concentric hamstring strength was assessed in a seated position using a Biodex System 4 (Biodex Medical Systems, Inc., Shirley, NY, USA), at an angular velocity of 90°/s with a range of motion of 0–90° of knee flexion (Fig. 1). Prior to the maximum attempts, a standardized warm-up consisting of ten minutes on a stationary bike, ten submaximal attempts at 50% and 75%, and one submaximal attempt at 90% of maximal effort was performed. For the maximal attempts, one repetition of maximum knee extension directly followed by knee flexion was performed with 40 s rest between each attempt. Three to four attempts were performed and the peak torque value in Newton meter (Nm) was registered in the Project ACL’s database. The Biodex has been reported to have high reliability for test-retest measures or peak torque, corresponding to an ICC of between 0.82 and 0.99 [19, 20].

**Fig. 2:**
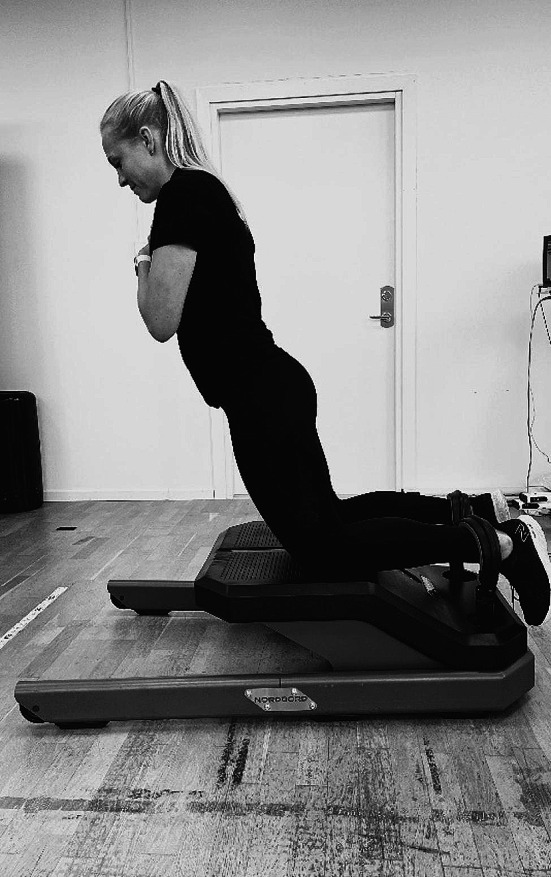
The eccentric Nordic hamstring exercise performed in the NordBord

Second, the eccentric hamstring strength was assessed in the Nordic hamstring position using a NordBord device (Vald Performance NordBord, Version 1.0; Australia) [21] (Fig. 2). Prior to the maximum attempts, a standardized warm-up consisting of two submaximal attempts at 50% of the maximum effort was performed. Two sets with three repetitions of maximum effort were performed in each set with two minutes rest between sets. Between each set, the patients had the opportunity to see the force curve on an iPad. The maximum force value in Newton (N) from a single repetition was registered in the registry database. The test has high test-retest reliability for measuring eccentric hamstring strength, corresponding to ICC values between 0.83 and 0.90 [21, 22].

In the present study, we reported the strength results as LSI and relative strength. The LSI was defined as the strength in relation to the uninjured limb and expressed as a percentage. Relative strength was defined as the peak torque or peak force for the involved limb normalized to body weight and expressed as Nm/kilogram (kg) in the Biodex test and N/kg in the NordBord test. The relative strength of the uninjured limb was not considered in the present study.

### Hop performance

Three hop tests were performed in the Project ACL’s test battery: the vertical hop, hop for distance and the 30 s side hop test. Hop tests were performed between the two different hamstring strength tests: i.e., (1) Biodex isokinetic strength test, (2) hop tests, and (3) NordBord eccentric strength test. Familiarization with two to three submaximal trials was allowed prior to the vertical hop and hop distance tests, and familiarization with ten hops was allowed prior to the 30 s side hop test. To standardize the hop tests, the patients were asked to hold their hands behind their backs during all tests. Patients performed three single maximal attempts in the vertical hop test. The time in the air from take-off to landing was converted to centimeters (cm) using the Muscle Lab (Ergotest Technology, Oslo, Norway). In the hop for distance, patients were allowed three to five maximum attempts of which the distance between toes at take-off to heel at a stable landing (i.e., not moving the foot, not letting go of hands behind the back, or support with the opposite foot towards the floor at landing) was measured in cm. The third hop test was the 30 s side hop test, in which patients were allowed one maximal attempt, instructed to hop as many times as possible past two lines 40 cm apart for 30 s. The total number of hops performed without touching a line was recorded. All the best hop results were entered into the Project ACL database. The hop tests used in Project ACL have a high ability to discriminate hop performance between the reconstructed and uninjured side in patients with ACL reconstruction, and have a high test-retest reliability in patients who have undergone ACL reconstruction, corresponding to ICC values between 0.85 and 0.97 [23].

Hop performance was considered as a symmetry between the reconstructed and uninjured sides (LSI) for all hop tests. In addition, the hop distance was normalized to body height (hop distance divided by body height [both cm]), as greater body height may influence the maximum hop distance in cm. In addition, the vertical and side hop tests were considered in absolute terms, that is, the height of the vertical hop in cm, and the total number of successful side hops without errors.

### Outcomes

The primary outcome was the correlation between hamstring strength LSI for (1) the NordBord and (2) the Biodex, with hop performance (both LSI and relative values) at 8 and 12 months in patients after ACL reconstruction treated with HT autografts. The secondary outcome was the correlation between relative hamstrings strength in (3) the NordBord and (4) the Biodex test and hop performance (both LSI and relative values) at 8 and 12 months in patients after ACL reconstruction treated with HT autografts. A sub-analysis was performed on patient sex for both the primary and secondary outcomes.

The time points of 8 and 12 months were chosen because they represent the timeframe in which patients are typically advised to undergo test batteries prior to returning to sports [12, 24].

### Statistical analysis

Descriptive statistics were calculated using means and standard deviations for parametric data, medians, and interquartile ranges for ordinal data, and counts and percentages for non-parametric data. The significance level was set at *p* < 0.05. To analyze the correlation between hamstring strength and hop performance, Pearson’s correlation coefficient was used and interpreted using the following reference values: 0.1–0.3 = weak, 0.4–0.6 = moderate, 0.7–0.9 = strong, and 1 = perfect [25]. Values between 0.3 to 0.4 were interpreted as weak to moderate, 0.6 to 0.7 as moderate to strong, and 0.9 to 1.0 as strong to perfect. Prior to correlation analysis, outliers were assessed using a scatter plot. No outliers were present that affected the results of the correlation analysis using Pearson’s correlation coefficient. In addition, the coefficient of determination (r^2^) was calculated to interpret the clinical relevance, that is, the proportion of variance in the dependent variable (hop performance) explained by an independent variable (hamstring strength). Based on a power analysis of the estimated correlation coefficient of 0.38 [26], a power of 80%, and a significance value of 0.05, 52 patients were required. The estimated correlation coefficient was based on a previous study investigating the correlation between hamstring strength and hop distance [26]. Statistical analyses were performed using Statistical Product and Service Solutions (IBM Corp. Released 2017. IBM SPSS Statistics for Windows, Version 25.0. Armonk, NY: IBM Corp.)

## Results

**Fig. 3:**
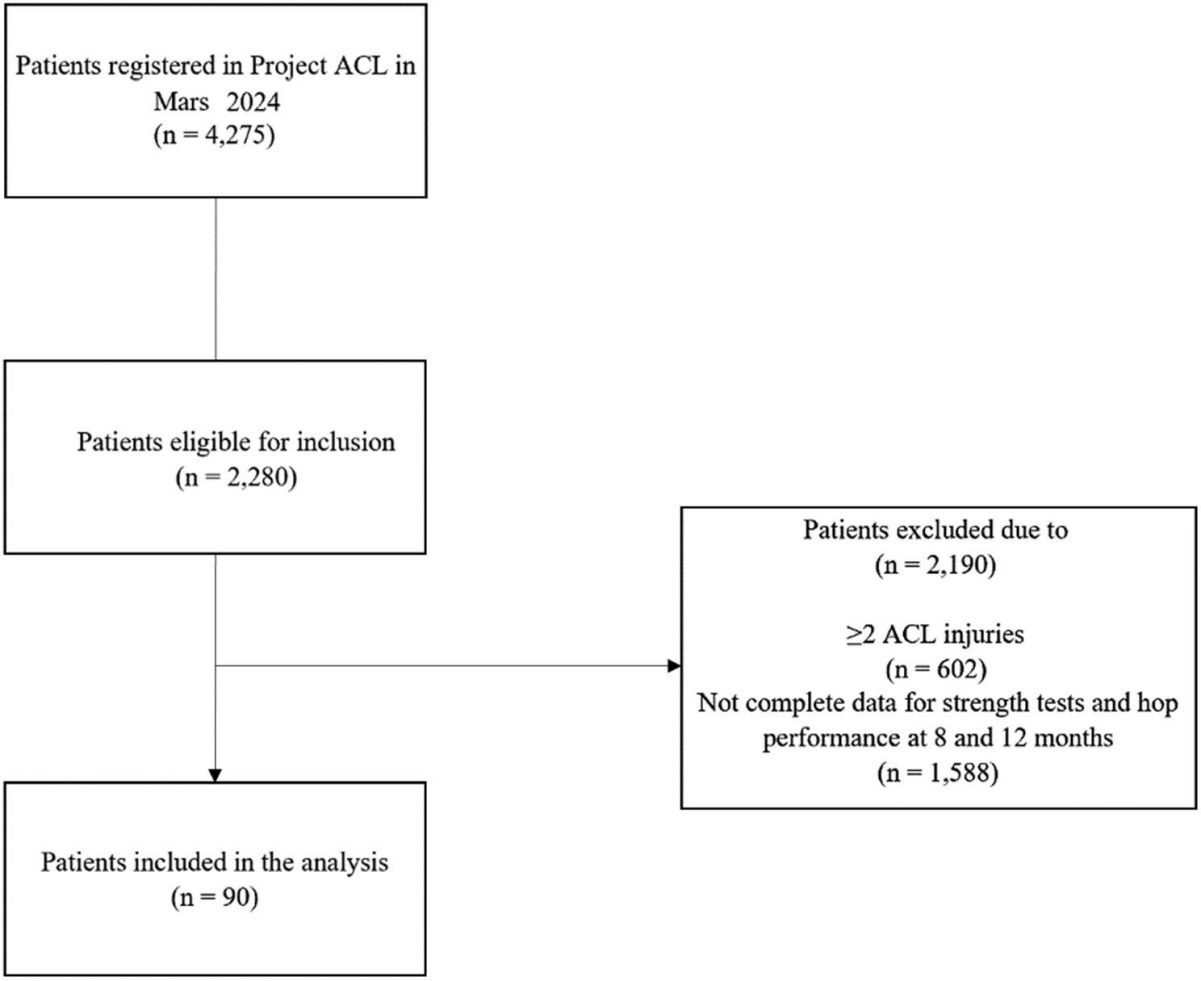
Flowchart of inclusion and exclusion

In total, 90 patients were included, of which 48 (53%) were women. The mean age at ACL reconstruction was 27.0 ± 8.0 years. The hamstring strength LSI was 95.1% ± 11.0% in the Biodex and 85.2% ± 13.5% in the NordBord at 8 months. At 12 months, the hamstring strength LSI was 96.1% ± 10.1% in the Biodex and 85.7% ± 12.6% in the NordBord. Men had significantly greater relative quadriceps and hamstring strength, vertical hop height, hop distance relative to height, and total number of side hops compared to women. Figure 3 displays the flowchart for inclusion and exclusion. Table 1 presents patient demographics, and Table 2 presents muscle strength and hop performance.

**Table 1:** Patient demographics. Mean presented with standard deviations

Patients, *n*	90
Women, n (%)	48 (53%)
Age, years	27.0 ± 8.0
Weight, kg	71.6 ± 12.3
Height, cm	174.3 ± 8.8
BMI, kg/m^2^	23.4 ± 2.9
Preinjury Tegner, median with minimum and maximum values	9 (6–10)
Days between injury and ACL reconstruction (median; IQR)	124.0 (84.5; 184.0)

BMI: Body mass index, cm: Centimeters, IQR: Interquartile range, kg: Kilograms, n: Number of patients, m: Meters; Tegner: Tegner Activity Scale

**Table 2:** Muscle strength and hop performance values at 8 and 12 months, presented for all, women, and men

	Sex	8 Months	12 Months
Concentric quadriceps strength (Biodex), LSI (%)	All	92.3% ± 9.3%	95.9% ± 9.3%
	W	91.6% ± 9.4%	95.2% ± 10.6%
	M	93.0% ± 9.3%	96.6% ± 7.6%
Relative concentric quadriceps strength (Biodex), Nm/kg	All	2.8 ± 0.6	2.9 ± 0.7
	W	**2.6 ± 0.4**	**2.6 ± 0.5**
	M	**3.0 ± 0.6**	**3.1 ± 0.7**
Concentric hamstring strength (Biodex), LSI (%)	All	95.1% ± 11.0%	96.1% ± 10.1%
	W	94.2% ± 10.9%	95.9% ± 9.5%
	M	96.1% ± 11.3%	96.3% ± 10.9%
Relative concentric hamstring strength (Biodex), Nm/kg	All	1.6 ± 0.4	1.6 ± 0.4
	W	**1.4 ± 0.3**	**1.4 ± 0.3**
	M	**1.7 ± 0.4**	**1.8 ± 0.4**
Eccentric hamstring strength (NordBord), LSI (%)	All	85.2% ± 13.5%	85.7% ± 12.6%
	W	84.3% ± 13.5%	83.8% ± 10.0%
	M	86.4% ± 13.5%	87.8% ± 14.8%
Relative eccentric hamstring strength (NordBord), N/kg	All	3.9 ± 1.2	4.0 ± 1.1
	W	**3.6 ± 0.9**	**3.7 ± 0.9**
	M	**4.2 ± 1.4**	**4.3 ± 1.3**
Vertical hop, LSI (%)	All	86.6% ± 13.7%	91.0% ± 14.1%
	W	86.8% ± 14.6%	91.4% ± 15.7%
	M	86.4% ± 12.8%	90.3% ± 12.2%
Vertical hop, cm	All	13.8 ± 4.3	15.1 ± 4.5
	W	**12.1 ± 3.9**	**13.1 ± 4.0**
	M	**15.8 ± 3.8**	**17.4 ± 4.0**
Hop for distance, LSI (%)	All	91.7% ± 9.1%	95.2% ± 9.4%
	W	92.7% ± 9.0%	94.8% ± 11.2%
	M	90.7% ± 9.3%	95.7% ± 6.8%
Hop for distance relative to height, distance/height in cm	All	0.72 ± 0.14	0.76 ± 0.14
	W	**0.69 ± 0.13**	**0.72 ± 0.14**
	M	**0.76 ± 0.13**	**0.80 ± 0.12**
Side hop, LSI (%)	All	90.5% ± 17.3%	96.3% ± 16.5%
	W	87.8% ± 20.1%	97.8% ± 18.0%
	M	93.7% ± 12.9%	98.3% ± 9.6%
Side hop, total number of hops	All	42 ± 18	48 ± 17
	W	**34 ± 16**	**42 ± 16**
	M	**50 ± 16**	**55 ± 15**

There were 48 women and 42 men participating at the 8- and 12-month follow-up. Mean presented with standard deviations. Bold numbers indicate a significant difference between women and men, *p* < 0.05. Kg: Kilogram, LSI: Limb symmetry index, M: Men, n: Number of patients, N: Newton, Nm: Newton meter, W: Women

### Correlations between hamstring strength LSI with hop performance at 8 and 12 months after ACL reconstruction

At 8 months after ACL reconstruction, the hamstring strength LSI in the NordBord test had significant weak, and weak to moderate positive correlations with vertical hop height, total number of side hops, and hop for distance relative to body height (*r* = 0.22–37, r^2^ = 4.8 − 13.7%). There were significant weak positive correlations between hamstring strength LSI in Biodex and hop for distance relative to height and total number of side hops (*r* = 0.24–25, r^2^ = 5.8–6.3%).

**Fig. 4:**
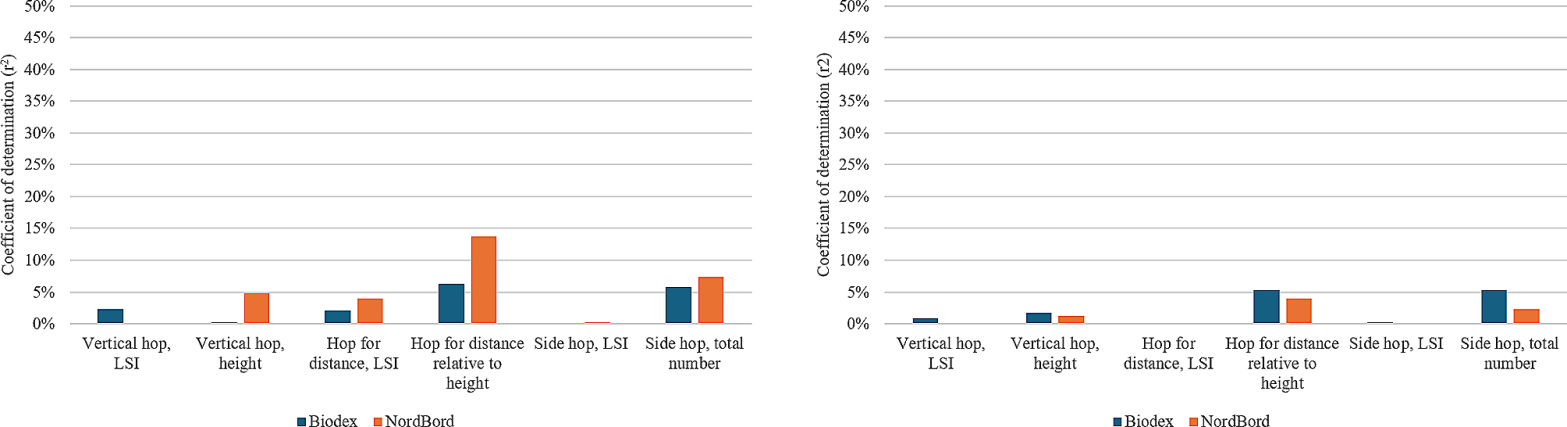
Coefficient of determination for hamstring strength limb symmetry index and hop performance at 8 months to the left, and 12 months to the right

At 12 months after ACL reconstruction, there were no significant correlations between hamstring strength LSI for NordBord and hop performance. The hamstring strength LSI in the Biodex had significant weak positive correlations with hop for distance relative to height and total number of side hops (*r* = 0.23, r^2^ = 5.3%) (Table 3; Fig. 4).

**Table 3:** Correlations between hamstring strength limb symmetry index for Biodex and NordBord with hop performance at the 8- and 12-month follow-up

	Biodex				NordBord			
	8 Months		12 Months		8 Months		12 Months	
	r	p	r	p	r	p	r	p
Vertical hop, LSI	-	n.s	-	n.s	-	n.s	-	n.s
Vertical hop, height	-	n.s	-	n.s	**0.22**	**0.04**	-	n.s
Hop for distance, LSI	-	n.s	-	n.s	-	n.s	-	n.s
Hop for distance relative to height	**0.25**	**0.02**	**0.23**	**0.03**	**0.37**	**< 0.001**	-	n.s
Side hop, LSI	-	n.s	-	n.s	-	n.s	-	n.s
Side hop, total number	**0.24**	**0.02**	**0.23**	**0.03**	**0.27**	**0.01**	-	n.s

Bold numbers indicate significant correlations. LSI: Limb symmetry index, n: Number of patients, n.s: non-significant, r: Correlation coefficient, p: p-value

### Correlations between hamstring LSI with hop performance specified by sex at 8 and 12 months after ACL reconstruction

For women, the hamstring strength LSI in the NordBord demonstrated significant weak to moderate positive correlations with hop performance, ranging from *r* = 0.32–0.47 (r^2^ = 10.2–22.1%), whereas no significant correlations were observed for men at 8 months after ACL reconstruction. The hamstring strength LSI for women in the Biodex demonstrated a significant weak to moderate positive correlation between hamstring strength LSI and total number of side hops (*r* = 0.31, r^2^ = 9.6%), whereas men did not have any significant correlations between hamstring strength LSI in the Biodex and hop performance (Supplementary Table 1).

At 12 months after ACL reconstruction, there was a significant weak to moderate correlation between hamstring strength LSI in the NordBord and hop distance relative to body height (*r* = 0.30, r^2^ = 9.0%) for women, whereas no significant correlation was observed for men. The hamstring strength LSI in the Biodex had weak positive correlation with total number of side hops (*r* = 0.29, r^2^ = 8.4%) for women, whereas no significant correlation was observed for men (Supplementary Table 1).

### Correlations between relative hamstring strength with hop performance at 8 and 12 months after ACL reconstruction

At 8 months after ACL reconstruction, significant weak and moderate positive correlations were observed for relative hamstring strength in the NordBord and LSI for hop distance, vertical hop height, hop for distance relative to height, and total number of side hops (*r* = 0.25–0.52, r^2^ = 6.3–27.0%). The relative hamstring strength in the Biodex had significant weak to moderate, and moderate positive correlations with LSI for side hop, vertical hop height, hop for distance relative to height, and total number of side hops (*r* = 0.30–0.59, r^2^ = 9.0–34.8%).

**Fig. 5:**
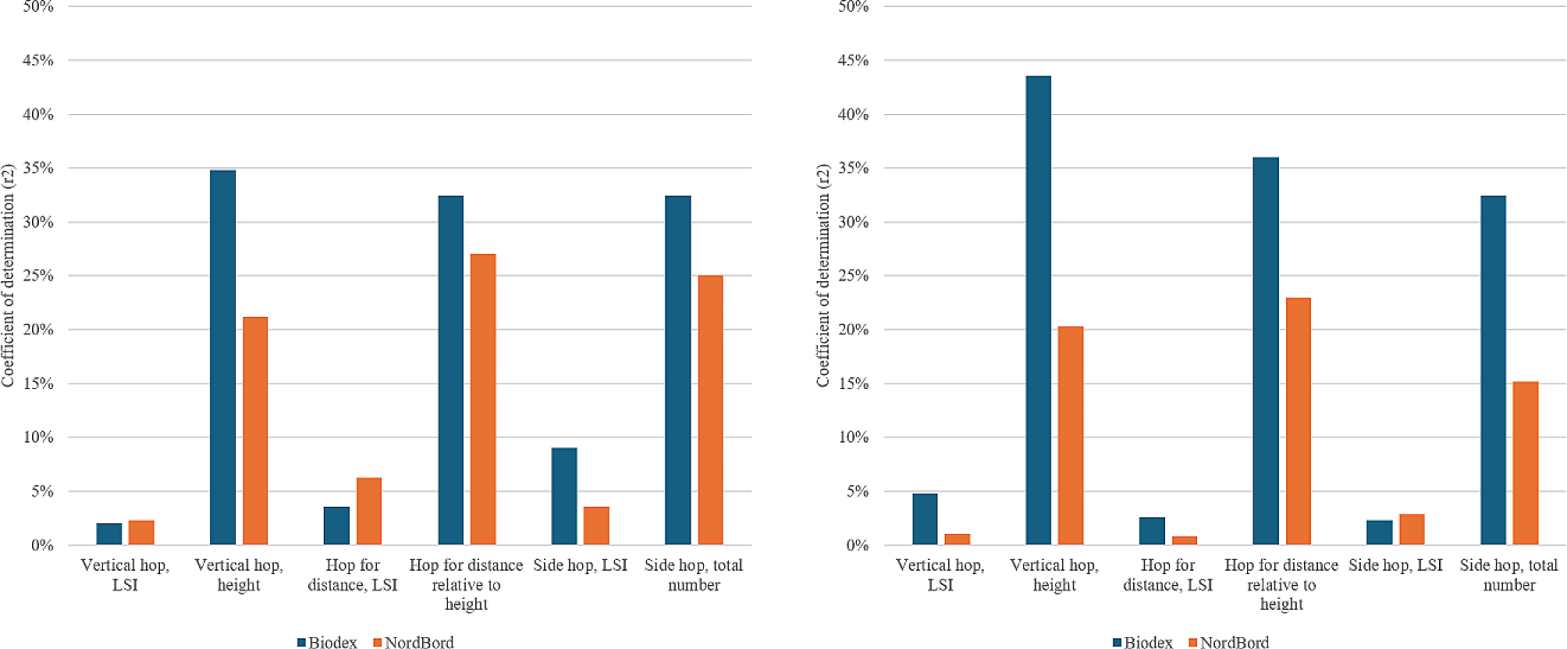
Coefficient of determination for relative hamstring strength and hop performance at 8 months to the left and 12-month to the right

At 12 months after ACL reconstruction, there were significant weak to moderate, and moderate positive correlations for relative hamstring strength in NordBord with total number of side hops, vertical hop height, and hop distance relative to height (*r* = 0.39–0.48, r^2^ = 15.2–23.0%). For the relative hamstring strength in the Biodex, there were significant weak, moderate, and moderate to strong positive correlations with the LSI for vertical hop, total number of side hops, vertical hop height, and hop distance relative to height (*r* = 0.22–0.66, r^2^ = 4.8–43.6%) (Table 4; Fig. 5).

**Table 4:** Correlations between relative hamstring strength and hop performance at the 8- and 12-month follow-up

	Biodex				NordBord			
	8 Months		12 Months		8 Months		12 Months	
	r	P	r	p	r	p	r	p
Vertical hop, LSI	-	n.s	**0.22**	**0.03**	-	n.s	-	n.s
Vertical hop, height	**0.59**	**< 0.001**	**0.66**	**< 0.001**	**0.46**	**< 0.001**	**0.45**	**< 0.001**
Hop for distance, LSI	-	n.s	-	n.s	**0.25**	**0.02**	-	n.s
Hop for distance relative to height	**0.57**	**< 0.001**	**0.60**	**< 0.001**	**0.52**	**< 0.001**	**0.48**	**< 0.001**
Side hop, LSI	**0.30**	**0.005**	-	n.s	-	n.s	-	n.s
Side hop, total number	**0.57**	**< 0.001**	**0.57**	**< 0.001**	**0.50**	**< 0.001**	**0.39**	**< 0.001**

Bold numbers indicate significant correlations. LSI: Limb symmetry index, n: Number of patients, n.s: non-significant, r: Correlation coefficient, p: p-value

### Correlations between relative hamstring strength with hop performance specified by sex at 8 and 12 months after ACL reconstruction

At 8 months after ACL reconstruction, women had significant weak to moderate positive correlations between the relative hamstring strength in the NordBord test and hop performance, ranging from *r* = 0.35–0.50 (r^2^ = 12.3–25.0%). For men, the relative hamstring strength in the NordBord test demonstrated significant moderate positive correlations with hop performance, ranging from *r* = 0.42–0.53 (r^2^ = 17.6–28.1%). Women had significant weak to moderate positive correlations between relative hamstring strength in the Biodex and hop performance, ranging from *r* = 0.36–0.48 (r^2^ = 8.4–23.0%). Men had significant weak to strong positive correlations between relative hamstring strength in the Biodex and hop performance, ranging from *r* = 0.31–0.60 (r^2^ = 9.6–36.0%) (Supplementary Table 2).

At 12 months after ACL reconstruction, women had significant weak to moderate positive correlations for relative hamstring strength in NordBord with hop performance, ranging from *r* = 0.34–0.44 (r^2^ = 11.6–19.4%). Men had significant weak to moderate positive correlations for relative hamstring strength in the NordBord with hop performance, ranging from *r* = 0.32–0.51 (r^2^ = 10.2–26.1%). Relative hamstring strength in the Biodex for women demonstrated significant moderate positive correlations with hop performance, ranging from *r* = 0.40–0.51 (r^2^ = 16.0–26.1%). Men had significant moderate to strong positive correlations between relative hamstring strength in Biodex and hop performance, ranging from *r* = 0.40–0.73 (r^2^ = 16.0–53.3%) (Supplementary Table 2).

## Discussion

Our main finding was that the relative hamstring strength in the Biodex explained 32.5–43.6% of the hop performance in vertical hop height, hop for distance relative to height, and the total number of side hops, whereas the relative hamstring strength in the NordBord explained 15.2–23.0% of the hop performance. Hence, at 8 and 12 months after ACL reconstruction, typical timepoints of return to sport, the relative hamstring strength assessed in a hip-flexed position may be a better indicator for hop performance in men and women reconstructed with HT autografts.

### Hamstring strength and hop performance

Recovery of hamstring strength after ACL reconstruction is considered an important part of the decision to return to sports [8, 27]. Comparison with the uninjured limb is frequently used as a proxy to interpret the recovery of the preinjury strength of the injured limb [7, 8]. An increase in the hamstring strength LSI does not necessarily reflect that the injured limb has become stronger but may be due to the loss of strength of the uninjured limb [7, 28]. The use of hamstring strength LSI lacks therefore information on the strength of the patients and whether the hamstrings are physically prepared for the demands of returning to strenuous knee activities. In our results, the hamstring strength LSI in both tests ranged from significant weak positive correlations to significant weak to moderate positive correlations with hop performance at 8 and 12 months after ACL reconstruction, accounting for 4.8–13.7% of the variance in hop performance. On the other hand, the relative hamstring strength of both the Biodex and NordBord at 8 and 12 months after ACL reconstruction ranged from significant weak positive correlations to significant moderate to strong positive correlations, accounting for 6.3–43.6% of the variance in hop performance. The discrepancy in the strength of the correlations and the greater proportions of variance in the correlations between the LSI and relative strength may indicate that the LSI approximates hamstring strength relative to the uninjured limb, whereas the relative hamstrings strength is a better indicator of how strong the patients are, resulting in a greater explained variance of hop performance. Collectively, regardless of how hamstring strength is assessed (i.e., NordBord or Biodex), the hamstring strength LSI has a weak relationship with hop performance, suggesting that relative hamstring strength may be of greater value for hop performance.

### The Biodex and NordBord test

The LSI in the NordBord displayed weak, and weak to moderate positive correlations with hop performance at 8 months, but no significant correlations at 12 months. In addition, the relative hamstring strength in the NordBord displayed moderate positive correlation with total number of side hops at 8 months, but only a weak to moderate positive correlation at 12 months. Weak to moderate positive correlations (*r* = 0.37–38) have been reported between relative hamstring strength in the NordBord and knee-self efficacy and psychological readiness at 8 months, but not at 12 months [14]. The NordBord test requires a supramaximal eccentric contraction and patients who displays greater relative hamstring strength values in the NordBord at 8 months, may be patients with a greater trust to their knee to perform activities, which may be reflected in greater hop performance at 8 months. Thus, the psychological aspect in the ability to trust their knee muscles to exert supramaximal force ‘earlier’ in the rehabilitation may be confounding. At the 12 months follow-up, the relative hamstring strength in the Biodex explained 32.5–43.6% of the hop performance in vertical hop height, hop for distance relative to height, and the total number of side hops, whereas the relative hamstring strength in the NordBord explained 15.2–23.0% of the hop performance at 12 months after ACL reconstruction. There may be several reasons for these observations, as the test performance differs in several ways; that is, the Biodex test was assessed in a seated position, unilaterally and concentrically, while the NordBord test was assessed in the Nordic hamstring position, bilaterally and eccentrically. After ACL reconstruction, patients may shift their load from the injured to the uninjured side during bilateral tasks [29]. To account for the possible limitation inferred by load shifting, the patients were shown the force curve between each set to be able to adjust in the case of unconsciously loading their uninjured side. Additionally, as the NordBord test was performed in the end, patients may have shifted their load to the uninjured side due to fatigue in the reconstructed leg. We applied a standardized protocol of two minutes of rest in between each set in the NordBord to facilitate recovery and reduce the influence of fatigue in the results. It can be questioned whether the discrepancy may be the result of different contraction types (concentric versus eccentric). However, previous research indicated that despite performing the Biodex eccentric, no significant association between the respective tests appears to exist [30]. Lastly, the NordBord was performed with the hip close to extension, while the seated Biodex test was performed with 85° of hip flexion. To perform hamstring exercises with an extended hip is associated with greater activation and hypertrophy for the semitendinosus muscle relative to the biceps femoris caput longum muscle [31, 32]. The discrepancy between semitendinosus and biceps femoris caput longum activation in knee flexion exercises with the hip close to extension may be explained by the moment arm of the biceps femoris caput longum decreasing with the hip close to extension, causing disadvantages in force production [33]. In summary, the greater coefficient of determination observed for the relative hamstring strength assessed by Biodex, may reflect, although speculative, an increased contribution from other muscles in the hamstring muscle group to compensate for an eventual altered morphological structure in the semitendinosus muscle and tendon [34].

### Differences in correlations according to sex

There are known sex differences with men being typically stronger than women, primarily due to larger muscle fibers [35]. In the muscle strength and hop performance results, men were stronger in relative terms for all strength and hop tests; thus, differences in relative hamstring muscle strength between the sexes may have influenced the correlation analysis between relative hamstring strength and hop performance when merged into one group. Nonetheless, both men and women had a similar overall pattern of significant correlations observed between hamstring strength and the hop performance tests. However, some correlations differed, with women having significant weak to moderate positive correlations between hamstring strength LSI in the Biodex with total number of side hops at 8 and 12 months, and significant weak to moderate positive correlations between the hamstring strength LSI in the NordBord with vertical hop height and hop for distance relative to height. Importantly, the observed significant correlations were weak, and due to the many analyses performed, the discrepancy between sexes may be the result of false-positive findings due to many analyses performed, that is, type I error.

### Methodological considerations

The use of correlation for statistical analysis implies limitations in the conclusions of the results. The use of correlation analysis can only state the relationship between two variables and not whether it is causative. Quadriceps muscle strength may be a confounding factor as the mean peak moment has been reported as a strong predictor for hop distance (r^2^ = 45%). On the other hand, mean peak moment of hamstring strength has also been reported to be a strong predictor for hop distance (r^2^ = 40%) [36]. In addition to quadriceps muscle strength, calf muscle strength [37] and self-efficacy in the task [38] may be confounding as well and were not accounted for in the analysis. The test order in Project ACL, i.e., (1) Biodex isokinetic strength test, (2) hop tests, and (3) NordBord eccentric strength test, may have contributed to fatigue for the latter hop tests and the NordBord test which could have affected the results. Furthermore, the sex-specific correlation analysis was underpowered as only 48 women and 42 men were available; thus, four more women and ten more men were needed for sufficient statistical power. Some of the correlations may have become significant due to the many statistical analyses performed (e.g., type I error); consequently, the strength of our analysis was to use the coefficient of determination together with the degree of correlation to value the clinical relevance of significant results.

### Future directions

Future studies should aim to investigate how NordBord strength in relation to body weight develops over a longer period of time and its association with functional performance, including cutting maneuvers. Most importantly, if hamstring strength in the NordBord test is associated with the occurrence of a second ACL injury upon return to sports activity.

## Conclusions

The relative hamstring strength explained 4.8–43.6% of hop performance, whereas hamstring strength LSI explained 4.8–13.7%. The relative hamstring strength in the Biodex test explained 32.5–43.6% of the hop performance in vertical hop height, hop for distance relative to height, and the total number of side hops, whereas the relative hamstring strength in the NordBord explained 15.2–23.0%. Thus, our findings suggest that relative hamstring strength, especially in the hip-flexed position may be a better indicator of hop performance at 8 and 12 months after ACL reconstruction in patients treated with HT autograft.

### Electronic supplementary material

Below is the link to the electronic supplementary material.

Supplementary Material 1

## Data Availability

The datasets generated and/or analysed during the current study are not publicly available but are available from the corresponding author on reasonable request.

## Abbreviations

ACL: Anterior Cruciate Ligament
Cm: Centimeters
HT: Hamstring Tendon
ICC: Intraclass Coefficient
LSI: Limb Symmetry Index
PROs: Patient–Reported Outcomes
S: Seconds
