# Supplementary material for: Relationship between hamstring strength and hop performance at 8 and 12 months after ACL reconstruction with hamstring tendon autografts
Source: BMC Sports Sci Med Rehabil. 2024 Jun 18;16:134. doi: 10.1186/s13102-024-00923-4 (PMC11184683; doi:10.1186/s13102-024-00923-4)
Supplement: Supplementary file 1 — Supplementary Material 1 [file 13102_2024_923_MOESM1_ESM.docx]

**Supplementary files**

Supplementary Table 1. Sex-specific correlations for hamstring strength limb symmetry index in the Biodex and NordBord with hop performance at 8- and 12-month follow-up.

|  |  | Biodex | | | | NordBord | | | |
| --- | --- | --- | --- | --- | --- | --- | --- | --- | --- |
|  | Sex | 8 Months | | 12 Months | | 8 Months | | 12 Months | |
|  |  | r | p | r | p | r | p | r | p |
| Vertical hop LSI | W | - | n.s | - | n.s | - | n.s | - | n.s |
|  | M | - | n.s | - | n.s | - | n.s | - | n.s |
| Vertical hop, height | W | - | n.s | - | n.s | **0.32** | **0.03** | - | n.s |
|  | M | - | n.s | - | n.s | - | n.s | - | n.s |
| Hop for distance LSI | W | - | n.s | - | n.s | - | n.s | - | n.s |
|  | M | - | n.s | - | n.s | - | n.s | - | n.s |
| Hop for distance relative to body height | W | - | n.s | - | n.s | **0.47** | **<0.001** | **0.30** | **0.04** |
|  | M | - | n.s | - | n.s | - | n.s | - | n.s |
| Side hop, LSI | W | - | n.s | - | n.s | - | n.s | - | n.s |
|  | M | - | n.s | - | n.s | - | n.s | - | n.s |
| Side hop, total number | W | **0.31** | **0.03** | **0.29** | **0.04** | - | n.s | - | n.s |
|  | M | - | n.s | - | n.s | - | n.s | - | n.s |

*There were 48 women and 42 men participating at the 8- and 12-month follow-up. Bold numbers indicate significant correlations.
LSI: Limb symmetry index, M: Men, n: Number of patients, n.s: non-significant, r: Correlation coefficient, p: p-value, W: Women.*

Supplementary Table 2. Sex-specific correlations for relative hamstring strength in the Biodex and NordBord with hop performance at 8- and 12-month follow-up.

|  |  | Biodex | | | | NordBord | | | |
| --- | --- | --- | --- | --- | --- | --- | --- | --- | --- |
|  | Sex | 8 Months | | 12 Months | | 8 Months | | 12 Months | |
|  |  | r | p | r | p | r | p | r | p |
| Vertical hop, LSI | W | - | n.s | - | n.s | - | n.s | - | n.s |
|  | M | - | n.s | **0.40** | **0.008** | - | n.s | - | n.s |
| Vertical hop, height | W | **0.36** | **0.01** | **0.51** | **<0.001** | **0.43** | **0.002** | **0.44** | **0.002** |
|  | M | **0.57** | **<0.001** | **0.64** | **< 0.001** | **0.42** | **0.006** | **0.34** | **0.03** |
| Hop for distance, LSI | W | - | n.s | - | n.s | **0.35** | **0.01** | - | n.s |
|  | M | - | n.s | - | n.s | - | n.s | - | n.s |
| Hop for distance relative to height | W | **0.48** | **<0.001** | **0.40** | **0.005** | **0.47** | **<0.001** | **0.39** | **0.006** |
|  | M | **0.60** | **<0.001** | **0.73** | **<0.001** | **0.53** | **<0.001** | **0.51** | **<0.001** |
| Side hop, LSI | W | - | n.s | - | n.s | - | n.s | - | n.s |
|  | M | **0.31** | **0.045** | - | n.s | - | n.s | - | n.s |
| Side hop, total number | W | **0.48** | **<0.001** | **0.51** | **<0.001** | **0.50** | **<0.001** | **0.34** | **0.02** |
|  | M | **0.49** | **0.001** | **0.49** | **0.001** | **0.44** | **0.003** | **0.32** | **0.04** |

*There were 48 women and 42 men participating at the 8-, and 12-month follow-up. Bold numbers indicate significant correlations.
LSI: Limb symmetry index, M: Men, n: Number of patients, n.s: non-significant, r: Correlation coefficient, p: p-value, W: Women.*
